# The Relationship between Hypertension and Periodontitis: A Cross-Sectional Study

**DOI:** 10.3390/jcm12155140

**Published:** 2023-08-05

**Authors:** Rossana Abud Cabrera Rosa, João Victor Soares Rodrigues, Marina Module Cláudio, João Paulo Soares Franciscon, Gabriel Mulinari-Santos, Thamiris Cirelli, Rafael Scaf de Molon, Valdir Gouveia Garcia, Leticia Helena Theodoro

**Affiliations:** 1 Department of Diagnostic and Surgery, School of Dentistry, São Paulo State University (UNESP), Araçatuba 16015-050, SP, Brazil; rossana@unisalesiano.com.br (R.A.C.R.); joao.vic.t@hotmail.com (J.V.S.R.); marinamodoloc@gmail.com (M.M.C.); joao.franciscon@unesp.br (J.P.S.F.); gabriel_mulinari@hotmail.com (G.M.-S.); rafael.molon@unesp.br (R.S.d.M.); 2 Center for Dental Assistance to Persons with Disabilities (CAOE), School of Dentistry, Araçatuba 16015-050, SP, Brazil; 3 Department of Dentistry, University Center of Associated School (UNIFAE), São João da Boa Vista 13870-377, SP, Brazil; thamiriscirelli@gmail.com; 4 Latin American Institute of Dental Research and Education (ILAPEO), Curitiba 80710-150, PR, Brazil; vg.garcia@uol.com.br

**Keywords:** periodontitis, hypertension, inflammation, observational study

## Abstract

Recent evidence suggests an association between hypertension and periodontitis, although the pathways and implications underlying both chronic conditions are still poorly understood. Therefore, the aim of this study was to evaluate the relationship between hypertension and periodontitis through an observational clinical study using periodontal, physical, and biochemical analyses in hypertensive and non-hypertensive individuals with periodontitis. A total of one hundred patients were divided into two groups. The first group was hypertensive patients with periodontitis. The second group was non-hypertensive patients with periodontitis. Periodontal parameters of probing depth, bleeding on probing, and clinical attachment level were evaluated. The systolic, diastolic, mean, and differential blood pressure were measured in the physical examination. In addition, body mass index and waist–hip ratio were verified. Biochemical tests for glycated hemoglobin, fasting blood glucose, estimated blood glucose, total cholesterol, high-density lipoprotein, creatinine, glutamate pyruvate transaminase, glutamic oxaloacetic transaminase, and C-reactive protein were evaluated. The data were submitted for statistical analysis (α = 0.05%). The results of this study demonstrated that patients with cardiovascular disease did not present with worse periodontal clinical parameters in the conditions studied. However, it is important to bear in mind that this cross-sectional study has some inherent limitations to its design; therefore, to study the relationship between hypertension and periodontitis further, an interventional randomized clinical trial should be conducted.

## 1. Introduction

Hypertension is defined as a chronic high blood pressure condition [1]. In addition to cardiovascular complications, it is associated with changes in collagen metabolism, elevated systemic inflammation, and increased oxidative stress [1]. Essential or primary hypertension is a multifactorial disorder related to the elevation of systolic and diastolic blood pressure greater than or equal to 140 mmHg and 90 mmHg, respectively [2]. Hypertension affects more than thirty percent of the adult population worldwide and the incidence of hypertensive individuals is estimated to increase [3]. The resistance to blood flow can increase the risk of many other disorders, such as kidney insufficiency, blindness, and aneurysm [4]. The impact of hypertension on different immunoinflammatory conditions, such as periodontitis, remains to be determined. 

Adherence to antihypertensive medications is a key component to controlling blood pressure levels, though less than half of individuals are diagnosed [5]. Additionally, more than 70% of hypertensive individuals cannot achieve systolic and diastolic blood pressure below 130 mmHg and 80 mmHg, respectively, even with antihypertensive drugs [6]. There are other aggravating risk factors for hypertension, such as alcohol abuse, physical inactivity, excessive sodium intake, and obesity [7]. The amplified systemic inflammation can also affect arterial dysfunction, and consequently, it increases the risk of developing hypertension [6]. In addition, hypertension can be predisposed in patients with other immunoinflammatory disorders, such as psoriasis, rheumatoid arthritis, systemic lupus erythematous, and likely periodontitis [8].

Periodontitis is a multifactorial chronic inflammatory disease predisposed by a dysbiosis of oral microbiota [9,10]. It is characterized by loss of the periodontium tissue surrounding teeth [9,10]. Periodontitis affects more than half of the world’s population according to previous epidemiological data [11]. Moreover, periodontitis is associated with lower socioeconomic power and hugely impacts the quality of life setting as a public health problem [12]. It has a direct relationship with other systemic conditions, for example, diabetes and rheumatoid arthritis [13], and smoking, which can increase the risk of cardiovascular disease and mortality [8,14].

Hypertension and periodontitis mutually release pro-inflammatory cytokines from immune-related cells, which both impact cardiovascular disease [15]. Exacerbated systemic inflammation can result in endothelial dysfunction following the onset of hypertension as well as atherosclerosis [16]. The endothelial dysfunction harmfully reduces periodontal vascularization and subsequently impairs periodontal health [16]. An earlier study using spontaneously hypertensive rats with periodontal disease demonstrated an increased rate of alveolar bone loss related to hypertension [17]. Interestingly, another animal study described a different mechanism of hypertension in monkeys via activation of the immune response induced by *Porphyromonas gingivalis* [18].

Treatment of periodontitis can improve oral health conditions, quality of life, and blood pressure since it inhibits the release of proinflammatory cytokines [4]. Nevertheless, data in the present literature are contradictory. Some authors support a direct relationship between hypertension and periodontal disease [5,8,15,19], and others did not find the stated association [20,21]. Recently, we have evaluated the effect of non-surgical periodontal treatment (NSPT) on periodontal clinical parameters and systemic blood pressure in patients with combined hypertension and periodontitis stage III grade B. The results of this study demonstrated that NSPT significantly reduced the blood level of C-reactive protein, although there were no significant reductions in the blood pressure parameters after NSPT [22]. Consequently, it is crucial to expand scientific investigations between hypertension and periodontitis. Therefore, the present cross-sectional study aimed to evaluate the relationship between hypertension and periodontitis through an observational clinical study using periodontal, physical, and biochemical analyses in hypertensive and non-hypertensive individuals with periodontitis. 

## 2. Materials and Methods

### 2.1. Experimental Design

The present study is a cross-sectional study with a convenience sample developed in the interval from 2018 to 2021. The project was submitted and approved by the Human Research Ethics Committee of the Faculty of Dentistry of Araçatuba, São Paulo State University, UNESP (CAAE: 92731918.0.0000.5420). All patients were individually informed about the nature of the study and signed an informed consent form.

### 2.2. Sample Calculation

Sample calculation used the formula of the occurrence of diseases in the population expectation proposed by the World Health Organization [23]. The sample size was calculated from a finite population of 135 patients who were being monitored in the extension project entitled “Periodontal medicine in academic training and in the transformation of social and preventive behaviors”, considering a prevalence reported in the scientific literature of 66% of hypertensive patients with periodontitis [16], with an accuracy of 5% and a confidence interval of 95%, idealizing a minimum sample of 89 individuals. The sample size calculation was performed using the public domain Epi-Info v7.2.1.0 (Centers for Disease Control and Prevention, Atlanta, GA, USA).

### 2.3. Sample Selection

**Figure 1:**
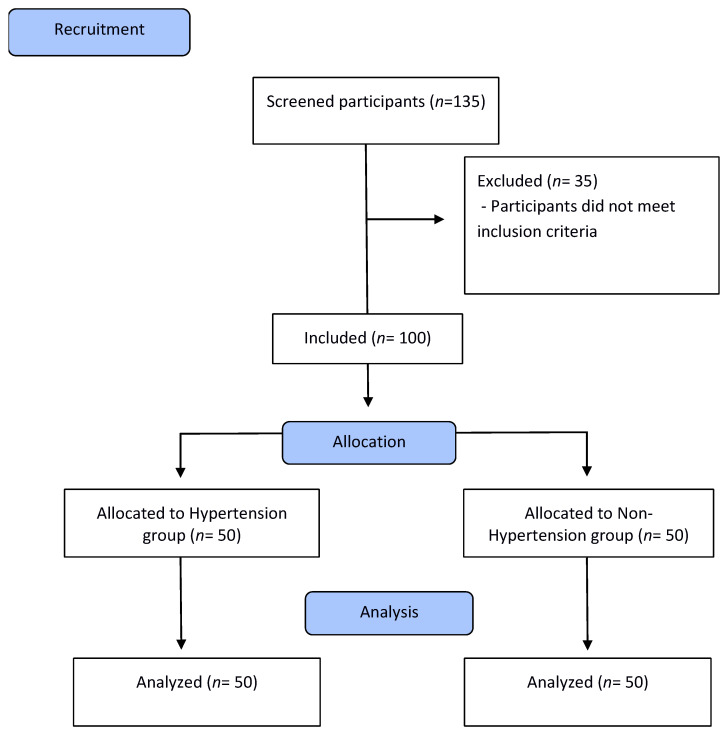
Flowchart of the study.

The total of participants included in the research was one hundred individuals who were recruited from the Oral and Systemic Health Clinic and Periodontics Clinic of the Dental School of Araçatuba, UNESP. They were divided into the hypertension group (*n* = 50), composed of hypertensive individuals with periodontitis, and the non-hypertension group (*n* = 50), of non-hypertensive individuals also with periodontitis (Figure 1).

The study inclusion criteria were as follows: individuals of both sexes; a diagnosis of periodontitis (stage II and III, grade B) was determined according to the recent periodontitis classification, with detectable clinical attachment loss at two or more non-adjacent interproximal sites with probing depths greater than 3 mm [9]. The determination of grade B periodontitis was based on radiographical assessment measuring the percentage of bone loss divided by the patients age in the worse periodontal scenario. All patients included in this study showed a moderate rate of disease progression less than 1, which characterizes grade B periodontitis; in addition, to be on antihypertensive treatment and have a previous medical diagnosis of arterial hypertension with systolic blood pressure greater than 140 mmHg or when diastolic blood pressure greater than 90 mmHg [2], only for patients in the hypertensive group. In the control group, systolic blood pressure should have been lower than 140 mmHg, diastolic blood pressure under 80 mmHg, and patients should not have taken or be currently taking antihypertensive medication. Patients with infectious diseases, diabetes mellitus, kidney or liver disorders, other cardiovascular diseases, cancer, and pregnant or lactating women were excluded [5].

All participants were scheduled individually for a unique session involving a physical examination and periodontal exam; the biochemical exams were then requested. In addition, a medical and dental history was performed to assess whether they were eligible to participate.

### 2.4. Physical Examination 

Participants were weighed and measured with a balance and a stadiometer to obtain body weight in kilograms and height in meters; the body mass index (BMI = weight [kg]/height^2^ [m^2^]) was then calculated [21]. The waist-to-hip ratio (WHR = waist [cm] ÷ hip [cm]) was obtained using a measuring tape at the waistline and hip, considering the iliac crest as a reference point with the limit values of 0.90 and 0.85 for men and women, respectively [24].

The blood pressure of each patient was measured via a digital electronic sphygmomanometer for non-invasive measurement (Welmy Balanças, Santa Bárbara d’Oeste, São Paulo, Brazil). All patients received a previous explanation of the measurement at least five minutes before in a calm environment. In addition, they were instructed to not talk and not have a full bladder. Additionally, the patient should not have practiced physical exercise and should not have ingested alcohol, coffee, or food in an interval of fewer than sixty minutes.

The blood pressure was measured with the patient seated, legs uncrossed, feet supported, back leaning, clothes not offering compression on the member, arm positioned at heart level and supported, with the palm facing upwards. The measurement was performed twice on both arms with an interval of one minute, and the highest value was recorded [2]. The following values were registered: systolic, diastolic, mean, and differential blood pressure, as in a previous study [2]. 

### 2.5. Periodontal Exam

In the clinical examination, in addition to the number of teeth, probing depth (PD), bleeding on probing (BOP), and clinical attachment level (CAL) examinations were also performed at all six sites of each tooth in the oral cavity. All clinical periodontal parameters were obtained with a millimeter periodontal probe (PCPUNC-15, Hu-Friedy, Chicago, IL, USA). The clinical examinations were performed by a calibrated and experienced examiner (JVSR). 

### 2.6. Biochemical Analysis 

A series of blood tests were requested for all participants. The exams requested were complete blood count, C-reactive protein (CRP), fasting blood glucose and estimated blood glucose, glycated hemoglobin (HbA1c), total cholesterol, high-density lipoprotein (HDL), glutamate-pyruvate transaminase (GPT), glutamic-oxaloacetic transaminase (GOT), and creatinine. The determination of positive CRP was characterized by levels equal to or above 6 mg/L; levels below was considered negative [25].

### 2.7. Examiner Calibration

Examiner calibration was performed before the periodontal analyses. For the calibration, one hundred and twenty sites were randomly selected in three patients. PD and CAL parameters were obtained on two separate occasions with a 7 day interval. Data were tabulated and submitted to the Kappa [26] agreement test, obtaining a value of 0.88.

### 2.8. Statistical Analysis

Demographic and clinical data were submitted for descriptive statistical analysis using GraphPad Prism v9.3.1 software (GraphPad Software, San Diego, CA, USA). Demographic variables between hypertensive and non-hypertensive groups were compared by the Student’s *t*-test for continuous variables (age, HBA1c, missing teeth, and blood pressure-related variables) and by the χ^2^ test for categorical variables (gender and CRP). The Mann–Whitney test was used to assess the association between continuous variables (biochemical data, physical examination, and periodontal exam). 

The association between each study variable and hypertension was estimated as the odds ratio (OR) and 95% confidence interval (CI) by age-adjusted multiple logistic regression analysis using STATA v17.0 software (Stata Corporation, College Station, TX, USA). In addition, multiple linear regression analysis was used to assess the relationship of each quantitative study variable independently with the presence of hypertension through the β coefficient and 95% CI.

## 3. Results

Table 1 presents the demographic characteristics of the participants. There was a statistical difference in the age distribution between the groups. The hypertension group presented a mean age of 60.4 ± 8.5 years and the non-hypertension group of 51.8 ± 8.6 years (*p* < 0.0001). There were no statistical differences in the gender of the groups. In periodontal parameters, the hypertension group had a lower number of teeth in the oral cavity when compared to the non-hypertension group (*p* = 0.016). The other periodontal parameters showed no differences. Interestingly, although there were no differences in the percentage of CAL among the CAL < 3 mm (6.6 and 4.9%), CAL 4–5 mm (64.9 and 68.8%), and CAL > 6 mm (27.9 and 25.6%) for the hypertensive and non-hypertensive groups, respectively, the CAL level of 4–5 mm presented with a trend to be statistically significant. This data correlated with a moderate severity of periodontitis for all the included patients. Higher percentages of HbA1c and fasting blood glucose were observed in the hypertension group, although without statistical significance (*p* = 0.014; *p* = 0.0253). The estimated blood glucose values showed no significant difference between groups (Table 1).

Biochemical data of total cholesterol, HDL, GPT, and creatinine presented no statistically significant differences between the groups. The differences were found particularly in the GOT (*p* = 0.043). Regarding the physical data, higher values of WRI were observed in the hypertensive group (*p* = 0.001). There was no difference in the BMI. In the evaluation of the CRP data, there were a greater number of patients who were positive for CRP in the hypertensive group when compared to the non-hypertensive group (*p* = 0.015; Table 1).

In the blood pressure analysis, higher values were found for the systolic pressure, mean blood pressure, and differential pressure in the hypertension group when compared to the non-hypertension group (*p* < 0.0001). There was no difference detected between the groups in the diastolic pressure data (Table 1).

The findings presented in Table 2 demonstrate the effect of hypertension associated with periodontitis through age-adjusted multiple logistic regressions. The data showed no association between the evaluated periodontal parameters and hypertension (*p* > 0.05). Regarding the glycemic profile, the regression indicated that hypertensive patients with periodontitis have higher levels of HbA1c (OR 2.39 95% CI 1.01–5.67, *p* = 0.04) and fasting glucose (OR 1.03 95% CI 1.01–1.07, *p* = 0.01). In addition, patients in the hypertension group had higher BMI rates (OR 1.12 95% CI 1.02–1.24, *p* = 0.01). Multiple logistic regression also indicated an association between increases in systolic pressure levels (OR 1.06 95% CI 1.02–1.09, *p* = 0.005), mean blood pressure (OR 1.08 95% CI 1.02–1.13, *p* = 0.008), and differential pressure (OR 1.05 95% CI 1.01–1.09, *p* = 0.04) in the hypertension group.

In Table 3, the multiple linear regression analysis indicated that patients with hypertension had fewer teeth (β = −0.21 95% CI −0.04–−0.004, *p* = 0.01). In addition, hypertension is also associated with higher levels of HbA1c (β = 0.16 95% CI 0.42–0.33, *p* = 0.04) and fasting blood glucose (β = 0.004 95% CI 0.002–0.011, *p* = 0.004), as well as higher BMI (β = 0.02 95% CI 0.006–0.38, *p* = 0.006). Similar to logistic regression, linear regression indicated that the hypertension group had higher systolic, mean, and differential blood pressure indices (β= 0.007 95% CI 0.003–0.02, *p* = 0.003; β = 0.02 95% CI 0.003–0.02, *p* = 0.007; β = 0.008 95% CI 0.008–0.02, *p* = 0.03).

## 4. Discussion

Hypertension and periodontitis are both chronic inflammatory conditions with a high incidence and prevalence in the adult population. Although the impact of these conditions can have many clinical consequences, there is still a lack of investigations considering them altogether. The main findings of this study were that patients with cardiovascular disease did not present with worse periodontal clinical parameters. Therefore, our study showed, in the conditions studied, that hypertensive patients did not show a negative impact in the periodontal tissues and alveolar bone. However, it is important to bear in mind that this cross-sectional study has some inherent limitations to its design; therefore, to study the relationship between hypertension and periodontitis further, an interventional randomized clinical trial should be conducted evaluating the effects of periodontal treatment on the biochemical parameters to come to the conclusion that there is no relationship between periodontitis and hypertension.

Tooth loss is an essential finding of periodontitis since periodontal disease progression results in the destruction of the periodontium [27]. Various previous cross-sectional studies have evaluated the association between tooth loss and systolic blood pressure [28,29,30,31]. A study with a multidisciplinary population of 1720 patients found a higher systolic blood pressure in edentulous individuals compared to patients with more than ten teeth [29]. Another study with more than four thousand patients affirmed an inversely proportional relationship between the number of teeth and blood pressure levels [30]. A previous study with 270 patients did not generate the same conclusions [28]. In the present study, a higher number of missing teeth in individuals with higher blood pressure rates was observed. However, the reasons for tooth loss in the included patients can be a consequence of periodontitis, as well as other reasons, such as periapical lesions, fractures, trauma, and caries [32]. Therefore, tooth loss cannot be stated only as consequence of periodontitis and hypertension since there was no correlation in the periodontal and blood pressure parameters.

A case-control study with 500 patients investigated the influence of periodontitis on blood pressure control [5]. The results showed an elevation of 3.36 mmHg and 2.16 mmHg, respectively, in the systolic and diastolic blood pressure of non-hypertensive individuals [5]. In addition, a systematic review and meta-analysis study demonstrated up to a 50% increased chance of hypertension in patients with moderate to severe periodontitis when compared with periodontal healthy patients [4]. Likewise, a retrospective cross-sectional analysis also confirmed a 20% increased probability of increased blood pressure to higher than 130 or 80 mmHg in adults with periodontitis compared with patients without periodontitis [33]. In contrast, an observational study analyzed the prevalence of hypertension and periodontitis, but it has not confirmed a direct association between periodontitis and high blood pressure [21], which corroborates our findings.

The relationship between hypertension and periodontitis is associated with a chronic immune–inflammatory disorder increased mainly by periodontal inflammation [28]. First, hypertension can intensify the activation of innate and adaptive immune cells, such as monocytes, macrophages, and T and B lymphocytes [28]. Molecules activated can damage the periodontal vasculature [5]. On the other hand, periodontopathogens can exacerbate the inflammation cascade by activating Th1 and Th17 lymphocytes, subsequently stimulating a hypertensive state by the inflammatory mechanism [34]. In addition, periodontitis increases levels of local and systemic inflammatory markers by promoting changes in neutrophil function, resulting in vascular and endothelial dysfunction [5]. In particular, the endothelial dysfunction disrupts the regulatory balance by decreasing nitric oxide and promoting an increase of interleukin-6 (IL-6), tumor necrosis factor (TNF-α), and blood levels of CRP [34].

The renin–angiotensin system, in addition to controlling blood pressure, acts on periodontal tissues [35]. A previous study revealed the formation of angiotensin II (Ang II) in samples of periodontal tissue from rats and humans [35]. In a model of induced periodontitis, Ang II was overexpressed in hypertensive animals [35]. In normotensive rats, there was a decrease in Ang II and a smaller amount of alveolar bone loss [35]. Other experimental studies have indicated the action of antihypertensive drugs on the renin–angiotensin system of periodontal tissues [36,37]. Antihypertensive blockers of Ang II receptors have been effective in inhibiting the production of IL-6, TNF-α, and receptor activator of the nuclear factor kappa-Β ligand, and consequently, they reduce alveolar bone loss in rats with experimental periodontitis [36,37]. Thus, antihypertensives might inhibit bone loss by periodontal disease in hypertensive conditions.

The CRP occurs primarily for an increased concentration of IL-6 [38]. Our findings confirmed a higher CRP level in the hypertensive group. However, there was no association between CRP and blood pressure levels in the logistic and linear regression analysis. The CRP analysis of this study was categorized as positive or negative considering a threshold of 6 mg/L, which it explains the lack of association in the regression analyses. A systematic review evaluated the presence of CRP in patients with periodontitis, showing strong evidence of elevated CRP in patients with periodontitis [38]. A large epidemiological study indicated the CRP rate as a mediator of these disorders since there elevated CRP was found in hypertension and periodontitis. Another study showed an upregulation of CRP in hypertensive patients compared to non-hypertensive patients, justifying the high systemic inflammation [39]. A case–control study also investigated the endothelial dysfunction by the persistent pro-inflammatory state from periodontitis, finding higher CRP rates in patients with severe periodontitis [40].

In the present study, the mean age of each group showed a more advanced age in individuals with hypertension. Thus, logistic and linear regression analyses were adjusted for age. Our data showed that there were no differences regarding periodontal and biochemical nor physical parameters when the data was adjusted for age. Therefore, the increased age in the hypertensive group was not an indicator of negative clinical periodontal outcomes. These results corroborate with the data in the literature, in which authors report a higher prevalence of hypertension in individuals with more advanced ages [15,34], although it is not possible to exclude age as a risk for arterial changes. Patients aged between 30 and 45 years with periodontitis had reduced arterial caliber, showing not only aging but also vascular changes [33,41]. The HbA1c and fasting blood glucose were higher in the hypertensive group; thus, a correlation between hypertension and higher glycemia was confirmed. However, patients with diabetes were excluded from sample, given the prevalence of hypertension in patients with impaired glucose metabolism [42].

Anthropometric data of BMI and WHR were higher in hypertensive patients. Nevertheless, logistic and linear regression analyses have not correlated hypertension and WHR. Regarding BMI, the hypertension group was 1.12 more likely to be obese with a BMI over 30 kg/m^2^. Obesity has been reported as a risk factor for hypertension and cardiovascular disease [43]. Additionally, a dietary study described a greater prevalence of hypertension in obese patients [44]. This study investigated the possible influence of 27 nutritional factors on blood pressure and it showed uncontrolled pressure related to high sodium intake [44]. Presently, obesity is linked with the progression of non-transmissible diseases, such as hypertension and diabetes mellitus type 2, both associated with a diet rich in refined foods, sedentary lifestyles, and increasing aging [8]. Hence, it is possible to establish a profile of a hypertensive patient with a higher BMI and glycaemia when compared to non-hypertensive patients [2].

The systolic but not diastolic blood pressure was elevated in the hypertensive group when compared to the non-hypertensive group. The correlation of hypertension with periodontitis was confirmed by the logistic and linear regressions. In addition, the blood pressure values registered in this research corroborate with the literature [2]. Observational and intervention studies point to a greater association of the systolic blood pressure with periodontitis than the diastolic blood pressure [4,33,45]. The influence of systemic inflammation on the diastolic blood pressure is still not clear, although this is not considered a strong predictor of cardiovascular disease [34]. A recent study also stated the causal association of periodontitis and hypertension by Mendelian randomization used as a test of the causal relationship between risk factors and various phenotypes [6]. This same study showed a reduction of 7.5 ± 10 mmHg in systolic blood pressure after the non-surgical periodontal treatment [6]. In addition, another recent study found a 2.3 to 3 mmHg lower systolic blood pressure in hypertensive patients with satisfactory periodontal health [33].

Although this study adds more knowledge to the current literature on hypertension and periodontitis, some important caveats should be mentioned. The cross-sectional nature of the study design does not allow causal inferences regarding the relationship between cardiovascular disease and periodontitis to be made, and the assessment of disease incidence. Due to the need for a large and heterogeneous study population in cross-sectional studies, it is important to conduct future observational studies including a wider number of individuals, as well as studies analyzing the effect of periodontal treatment to assess the possible association between hypertension and periodontitis.

## 5. Conclusions

The results of this study demonstrated that patients with cardiovascular disease did not present with worse periodontal clinical parameters in the conditions studied. However, it is important to bear in mind that this cross-sectional study has some inherent limitations to its design; therefore, to study the relationship between hypertension and periodontitis further, an interventional randomized clinical trial should be conducted. Therefore, it is suggested to conduct future observational studies including a larger number of individuals, as well as studies analyzing the effect of periodontal treatment to assess the possible association between hypertension and periodontitis.

## Figures and Tables

**Table 1:** Comparison of demographic, periodontal, biochemical, physical data, and blood pressure levels.

	Hypertension	Non-Hypertension	*p*-Value
	*n* = 50	*n* = 50	
**Demographic Characteristics**			
Age, Mean (±SD)	60.4 (±8.5)	51.8 (±8.6)	<0.0001
Gender, *n* (%)			
Man	25 (50.0)	21 (42.0)	ns
Woman	25 (50.0)	29 (58.0)	ns
**Periodontal Parameters, Median (IQR)**			
Number of teeth	22.5 (15.7–26.0)	26.0 (22.0–28.0)	0.016
BOP (% of sites)	35.5 (24.8–52.4)	44.4 (26.7–62.8)	ns
PD ≤ 4 mm (% of sites)	94.5 (86.4–98.1)	95.1 (82.0–98.3)	ns
PD ≥ 5 mm (% of sites)	5.4 (1.9–13.6)	4.9 (1.1–18.1)	ns
CAL ≤ 3 mm (% of sites)	6.6 (2.0–12.3)	4.9 (1.1–18.8)	ns
CAL = 4–5 mm (% of sites)	64.9 (50.3–76.8)	68.8 (35.9–80.6)	ns
CAL ≥ 6 mm (% of sites)	27.9 (16.4–39.2)	25.6 (16.2–40.1)	ns
**Biochemical and Physical Data, Median (IQR)**			
HbA1c (%)	6.0 (5.6–6.4)	5.6 (5.2–6.1)	0.0104
Fasting Blood Glucose (mg/dL)	102.0 (93.0–117.5)	95.2 (84.5–108.8)	0.0253
Estimated Blood Glucose (mg/dL)	118.5 (102.0–128.1)	111.1 (99.3–124.0)	ns
Total cholesterol (mg/dL)	170.0 (147.5–222.0)	186.0 (144.0–217.0)	ns
HDL cholesterol (mg/dL)	53.0 (31.9–57.0)	45.3 (37.1–57.1)	ns

Abbreviations: BOP, bleeding on probing; PD, probing depth; CAL, clinical attachment level; HbA1c, glycated hemoglobin; HDL, high density level of cholesterol; GPT, glutamate pyruvate transaminase enzyme; GOT, glutamic oxaloacetic transaminase enzyme; BMI, body mass index; IQR, interquartile range (25–75% percentile); SD, standard deviation; ns, non-significance. The categorical sex and C-reactive protein intergroup comparisons were made using the Chi squared test. For age and blood pressure levels, periodontal parameters data were analyzed using the *t*-test (parametric variables) and laboratorial and physical data were analyzed using the Mann–Whitney test (non-parametric variables).

**Table 2:** Multiple logistic regression adjusted for age. Adjusted ORs with 95% CIs were estimated by multiple logistic regression models after controlling for age.

**Risk Factor**	**Adj OR (95% CI)**	***p*** **-Value**
**Demographic Characteristics**		
Gender	0.76 (0.30–1.92)	0.46
**Periodontal Parameters**		
Number of teeth	0.97 (0.89–1.06)	0.46
BOP	0.99 (0.96–1.01)	0.18
PD ≤ 4 mm	1.03 (0.97–1.07)	0.32
PD ≥ 5 mm	0.98 (0.94–1.02)	0.32
CAL ≤ 3 mm	0.97 (0.93–1.01)	0.17
CAL = 4–5 mm	1.02 (0.99–1.04)	0.05
CAL ≥ 6 mm	0.96 (0.93–1.01)	0.05
**Biochemical and Physical Data**		
HbA1c (%)	2.39 (1.01–5.67)	0.04
Fasting Blood Glucose (mg/dL)	1.03 (1.01–1.07)	0.01
Estimated Blood Glucose (mg/dL)	1.02 (0.99–1.05)	0.11
Total cholesterol (mg/dL)	0.99 (0.98–1.01)	0.99
HDL cholesterol (mg/dL)	1.01 (0.93–1.09)	0.79
C-reactive protein	6.31 (0.71–5.62)	0.09
Creatinine (mg/dL)	0.68 (0.08–5.75)	0.72
GPT	1.02 (0.99–1.05)	0.23
GOT	1.05 (0.98–1.11)	0.16
Waist/Hip Ratio (cm)	27.1 (0.12–6.15)	0.24
BMI (Kg/m^2^)	1.12 (1.02–1.24)	0.01
**Blood Pressure Variables**		
Systolic blood pressure	1.06 (1.02–1.09)	0.005
Diastolic blood pressure	1.05 (0.99–1.09)	0.09
Mean blood pressure	1.08 (1.02–1.13)	0.008
Differential blood pressure	1.05 (1.01–1.09)	0.04

Abbreviations: BOP, bleeding on probing; PD, probing depth; CAL, clinical attachment level; HbA1c, glycated hemoglobin; HDL, high density level of cholesterol; GPT, glutamate pyruvate transaminase enzyme; GOT, glutamic oxaloacetic transaminase enzyme; BMI, body mass index.

**Table 3:** Regression coefficient and 95% CI for effects of demographic characteristics, periodontal parameters, biochemical and physical data, and blood pressure variables (performed by multiple linear regression analysis). Adjusted β with 95% CIs were estimated by multiple logistic regression models after controlling for age.

**Risk Factor**	**Adjusted β (95% CI)**	***p*** **-Value**
**Demographic Characteristics**		
Gender	−0.05 (−0.25–0.14)	0.59
**Periodontal Parameters**		
Number of teeth	−0.21 (−0.04–−0.004)	0.01
BOP	−0.003 (−0.009–0.002)	0.18
PD ≤ 4 mm	0.004 (−0.004–0.01)	0.31
PD ≥ 5 mm	−0.004 (−0.13–0.004)	0.30
CAL ≤ 3 mm	−0.005 (−0.01–0.002)	0.19
CAL = 4–5 mm	0.003 (0.001–0.008)	0.06
CAL ≥ 6 mm	−0.007 (−0.014–−0.0001)	0.05
**Biochemical and Physical Data**		
HbA1c (%)	0.16 (0.42–0.33)	0.04
Fasting Blood Glucose (mg/dL)	0.004 (0.002–0.011)	0.004
Estimated Blood Glucose (mg/dL)	0.005 (−0.00–0.01)	0.10
Total cholesterol (mg/dL)	−0.000 (−0.002–0.003)	0.97
HDL cholesterol (mg/dL)	0.003 (−0.02–0.03)	0.82
Creatinine (mg/dL)	−0.81 (−0.53–0.37)	0.72
GPT	0.004 (−0.002–0.11)	0.21
GOT	0.008 (−0.002–0.02)	0.12
Waist/Hip Ratio (cm)	0.66 (−0.38–1.73)	0.21
BMI (Kg/m^2^)	0.02 (0.006–0.38)	0.006
**Pressure Variables**		
Systolic blood pressure	0.007 (0.003–0.02)	0.003
Diastolic blood pressure	0.008 (−0.002–0.18)	0.12
Mean blood pressure	0.02 (0.003–0.02)	0.007
Differential blood pressure	0.008 (0.008–0.02)	0.03

Abbreviations: BOP, bleeding on probing; PD, probing depth; CAL, clinical attachment level; HbA1c, glycated hemoglobin; HDL, high density level of cholesterol; GPT, glutamate pyruvate transaminase enzyme; GOT, glutamic oxaloacetic transaminase enzyme; BMI, body mass index.

## Data Availability

Data generated in this research project is available by contacting the last author of this paper via email. It is stored electronically as Excel worksheets.
